# Clinical validation of the Harmonised Cognitive Assessment Protocol (HCAP) in a Northern Irish population

**DOI:** 10.1002/alz70860_102313

**Published:** 2025-12-23

**Authors:** Claire Potter, Calum Marr, Leeanne O'Hara, Charlotte Neville, Michael McAlinden, Frank Kee, David R Weir, Bernadette McGuinness

**Affiliations:** ^1^ Queen's University Belfast, Belfast, Northern Ireland, United Kingdom; ^2^ Northern Ireland Clinical Research Network, Belfast Health and Social Care Trust, Belfast, Northern Ireland, United Kingdom; ^3^ University of Michigan, Ann Arbor, MI, USA

## Abstract

**Background:**

The Harmonised Cognitive Assessment Protocol (HCAP), first developed in the United States is a neurocognitive interview and assessment battery used in longitudinal studies of ageing around the world. It aims to assign participants into three distinct categories, cognitively normal, cognitively impaired not demented and likely dementia. A clinical validation study of the HCAP interview was completed in a memory clinic cohort recruited from Belfast, Northern Ireland (NI); the first validation of HCAP in a western clinical population.

**Methods:**

Participants aged 65 years and older, community dwelling without severe comorbid health or severe sensory issues were identified. Eighty participants were recruited from memory clinics in the Belfast Health and Social Care Trust (BHSCT) or the Ulster Independent Clinic (UIC) with a physician made diagnosis of mild cognitive impairment (MCI) (*n* = 40) or dementia (*n* = 40). Forty participants self‐reporting no cognitive decline were recruited from the NIHR's Join Dementia Research (JDR). Blinded to their clinical status, the research team then complete the HCAP interview including a family friend interview to ascertain functional status. Based on an algorithm developed within the HCAP international network, participants will be assigned one of the three clinical categories. Correlation between memory clinic diagnosis and HCAP assigned clinical category will be determined. Ethical approval was granted by the Office for Research Ethics Committee Northern Ireland (ORECNI) 22/NI/0007.

**Result:**

Recruitment, as of January 2025 is 91% with an anticipated completion date of March 2025. To date, 45.7% participants are male, mean age is 76.8 years (SD 6.1). The mean Mini‐Mental State Examination (MMSE) is 21.9 (SD 5.3). 42.5% participants with MCI or dementia were recruited from the BHSCT clinic and the rest recruited from UIC. All participants have completed all aspects of the interview, including nominating a family member or friend allowing planned statistical analysis to determine correlation with memory clinic diagnosis when recruitment is completed.

**Conclusion:**

The HCAP interview comprises a battery of cognitive assessments and a detailed family friend interview to estimate a clinical category based on cognitive status. The NI study is the first validation study of HCAP in a western clinical population.

